# 2021 CAEP Acute Atrial Fibrillation/Flutter Best Practices Checklist

**DOI:** 10.1007/s43678-021-00167-y

**Published:** 2021-08-12

**Authors:** Ian G. Stiell, Kerstin de Wit, Frank X. Scheuermeyer, Alain Vadeboncoeur, Paul Angaran, Debra Eagles, Ian D. Graham, Clare L. Atzema, Patrick M. Archambault, Troy Tebbenham, Andrew D. McRae, Warren J. Cheung, Ratika Parkash, Marc W. Deyell, Geneviève Baril, Rick Mann, Rupinder Sahsi, Suneel Upadhye, Erica Brown, Jennifer Brinkhurst, Christian Chabot, Allan Skanes

**Affiliations:** 1 Department of Emergency Medicine, University of Ottawa, Ottawa, ON Canada; 2 Ottawa Hospital Research Institute, Ottawa, ON Canada; 3 Department of Emergency Medicine, University of British Columbia, Vancouver, BC Canada; 4 Department of Emergency Medicine, Queen’s University, Kingston, ON Canada; 5 Department of Medicine, McMaster University, Hamilton, ON Canada; 6 Division of Emergency Medicine, McMaster University, Hamilton, ON Canada; 7 Division of Emergency Medicine, University of Toronto, Toronto, ON Canada; 8 Institute for Clinical Evaluative Sciences, Toronto, ON Canada; 9 Department of Emergency Medicine, University of Calgary, Calgary, AB Canada; 10 Department of Family Medicine and Emergency Medicine, Université Laval, Quebec, QC Canada; 11 Université de Montréal, Montreal, QC Canada; 12 Department of Emergency Medicine, Montreal Heart Institute, Montreal, QC Canada; 13 Hôpital de Granby, Granby, QC Canada; 14 Peterborough Regional Health Centre, Peterborough, ON Canada; 15 Trillium Health Partners, Mississauga Hospital, Mississauga, ON Canada; 16 Division of Emergency Medicine, Department of Family Medicine, McMaster University, Hamilton, ON Canada; 17 St. Mary’s General Hospital, Kitchener, ON Canada; 18 Division of Cardiology, Terrence Donnelly Heart Centre, St Michael’s Hospital, University of Toronto, Toronto, ON Canada; 19 Division of Cardiology, Dalhousie University, Halifax, NS Canada; 20 Heart Rhythm Services, Division of Cardiology, University of British Columbia, Vancouver, BC Canada; 21 Quebec City, QC Canada; 22 Division of Cardiology, Western University, London, ON Canada; 23 Clinical Epidemiology Unit, The Ottawa Hospital, F657, 1053 Carling Avenue, Ottawa, ON K1Y 4E9 Canada

## A. Assessment and risk stratification

### 1. Is AF/AFL with rapid ventricular response a primary arrhythmia or secondary to medical causes?

A. Rapid rate secondary to medical causes (usually in patients with pre-existing/permanent AF) e.g., sepsis, bleeding, PE, heart failure, ACS, etc.:
  o Investigate and treat underlying causes aggressively
  o Cardioversion may be harmful
  o Avoid aggressive rate control
B. Primary arrhythmia, e.g., sudden onset of AF/AFL

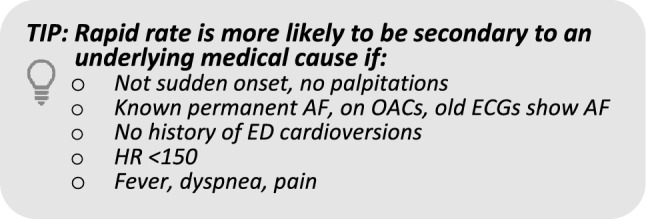


### 2. Is the patient unstable?

- Instability due to acute primary AF/AFL is uncommon, except for AF with rapid ventricular pre-excitation (WPW):
  o *Hypotension*: SBP < 90 mmHg, or signs of shock (e.g., altered mental status)
  o *Cardiac ischemia*: ongoing severe chest pain or marked ST depression (> 2 mm) on ECG despite therapy
  o *Pulmonary edema*: significant dyspnea, crackles, and hypoxia
- Treat unstable patient:
  o Urgent electrical CV if onset < 48 h or WPW
  o Consider trial of rate control if onset > 48 h

### 3. Is it safe to cardiovert this patient with primary AF/AFL?

- When it is safe, rhythm control is usually preferable to rate control: patient quality of life, shorter length of stay, fewer hospital resources
- It is safe to cardiovert if:
  A. The patient has been adequately anticoagulated for a minimum of 3 weeks, OR
  B. The patient is not adequately anticoagulated for > 3 weeks, has no history of stroke or TIA, AND does not have valvular heart disease, AND:
    1. Onset < 12 h ago, OR
    2. Onset 12—48 h ago and there are <2 of these CHADS-65 criteria (age ≥ 65, diabetes, hypertension, heart failure), OR
    3. Negative for thrombus on transesophageal echocardiography
- Consider delaying cardioversion if recent history of frequent palpitations
- Rate control acceptable, per patient and physician preference
  o e.g. older patients who are minimally symptomatic with a mildly elevated HR

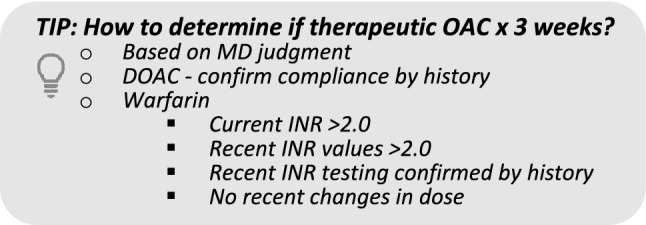


## B. Rate and rhythm control

### 4. Rate control for patients for whom cardioversion is unsafe

- Calcium channel- and beta-blockers considered first line:
  o If patient already taking oral calcium channel- or beta- blocker, choose same drug group first
  o If difficulty achieving adequate rate control**,** consider using the other first-line agent, IV digoxin, or cardiology consultation
- Calcium channel blocker:
  o Avoid if acute heart failure or known LV dysfunction (POCUS may be helpful)
  o Diltiazem 0.25 mg/kg IV over 10 min; repeat q15-20 min at 0.35 mg/kg up to 3 doses
  o Start 30–60 mg PO within 30 min of effective IV rate control
  o Discharge on 30-60 mg QID or Extended Release 120–240 mg once daily
- Beta blocker:
  o Metoprolol 2.5–5 mg IV over 2 min, repeat q15–20 min up to 3 doses
  o Start 25–50 mg PO within 30 min of effective IV rate control
  o Discharge on 25–50 mg BID
- Digoxin is second line, as slow onset:
  o 0.25–0.5 mg loading dose, then 0.25 mg IV q4–6 h to a max of 1.5 mg over 24 h; caution in renal failure
  o Consider first line if hypotension or acute HF
- Heart rate target: < 100 bpm at rest, < 110 walking

### 5. Rhythm control

- Either pharmacological or electrical cardioversion acceptable, per patient and physician preference:
  o Consider previous episodes; if one doesn’t work, try the other
- Pre-treatment with rate control agents not recommended – ineffective and delays treatment
- Pharmacological cardioversion:
  o Procainamide IV—15 mg/kg in 500 ml NS over 60 min, maximum 1500 mg
    Avoid if SBP < 100 mm Hg or QTc > 500 ms
    Interrupt infusion if BP drops or QRS lengthens visibly (e.g., > 30%)
    Check QTc after conversion
  o Amiodarone IV not recommended—slow, low efficacy
  o Less commonly used options include: vernakalant IV, ibutilide IV, propafenone PO and flecainide PO
- Electrical cardioversion
  o Setup—minimum 2 staff (RN/RRT; RN/RN), 2^nd^ physician ideal
  o Procedural sedation per local practice—e.g., Fentanyl, Propofol
  o Pad/paddle position—either antero-lateral or antero-posterior acceptable:
    Avoid sternum, breast tissue
    If failure, apply pressure with paddles, try the other position
  o Start with 150–200 J synchronized—avoid starting with low energy level
- Many patients can be discharged as soon as 30 min after conversion if treated with IV procainamide or ECV

### 6. Rapid ventricular pre-excitation (WPW)

- Urgent electrical CV usually required
- Procainamide IV if stable
  o AV nodal blocking agents contraindicated: digoxin, calcium channel-, beta-blockers, adenosine, amiodarone

## C. Stroke prevention

### 7. Who requires anticoagulation?

- Antithrombotic therapy prescribed at discharge is for long-term stroke prevention
- For OAC contraindications see the ‘McMaster Checklist’
- If CHADS-65 positive (any of age ≥ 65, diabetes, hypertension, heart failure, stroke/TIA) initiate OAC prior to discharge; consider shared decision making to include patients’ preferences with regards to risks and benefits:
  o DOACs preferred over warfarin
  o Use warfarin (*DOACs contraindicated)* if mechanical valve, moderate-severe mitral stenosis, severe renal impairment (*CrCl* < *30 ml/min*)
  o If stable CAD, discontinue ASA
  o If CAD with other anti-platelets or recent PCI < 12 months, consult cardiology
- If CHADS-65 negative, OAC might be considered for a 4-week period after careful consideration of risks and benefits and a shared decision-making process with the patient; ensure patient is aware anticoagulation will be discontinued after 4 weeks
- CHADS-65 negative and stable coronary, aortic, or peripheral vascular disease, ensure patient is on ASA 81 mg daily
  o Patients already taking anti-platelet agents require follow-up with cardiology
- If TEE-guided CV, must initiate DOAC immediately × 4 weeks
  o If warfarin, need LMW heparin bridging
- Patients who convert spontaneously before ED treatment should generally be prescribed OAC according to the CHADS-65 criteria

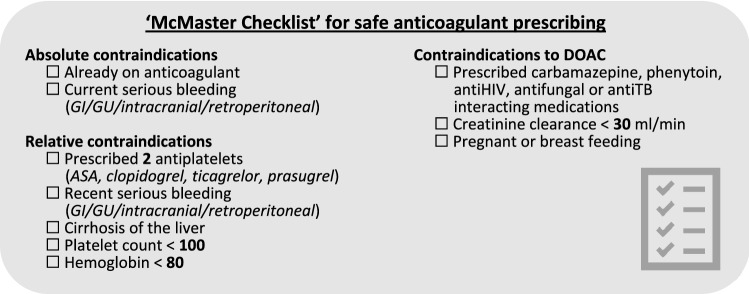

### 8. DOACs and warfarin

- See *Thrombosis Canada* App for details; avoid in pregnancy, breastfeeding
- Consult nephrology or thrombosis if CrCl < 30 ml/min
- Provincial formularies may require Limited Use codes, e.g. failure of warfarin or INR monitoring not possible:
  o Dabigatran—150 mg BID; use 110 mg BID if age > 80 years, or > 75 years with bleeding risk
  o Rivaroxaban—20 mg daily; use 15 mg daily if CrCl 30–49 ml/min
  o Apixaban—5 mg BID; use 2.5 mg BID if two of: (1) serum creatinine > 133 umol/L, (2) age > 80 years, or (3) body weight < 60 kg
  o Edoxaban—60 mg daily; use 30 mg daily if CrCl 30–50 ml/min or weight < 60 kg; important drug interactions
- Warfarin
  o Initiate warfarin: 5 mg daily; (1–2 mg daily if frail, low weight, Asian descent):
    Heparin bridging not required unless TEE-guided CV
    Arrange for INR blood test and review after 3 or 4 doses of warfarin. Subsequent warfarin doses should be communicated to patient on the day of the INR test

## D. Disposition and follow-up

### 9. Admission to hospital

- Patients rarely require hospital admission for uncomplicated acute AF/AFL unless they:
  o Are highly symptomatic despite adequate treatment
  o Have ACS with significant chest pain, troponin rise, and ECG changes
    No need to routinely measure troponin, small demand rise expected
  o Have acute heart failure not improved with ED treatment

### 10. Follow-up issues

- Recommend physician follow-up < 7 days if new warfarin or rate control meds
- Recommend cardiology / internal medicine follow-up in 4–6 weeks if not already followed or if new medications prescribed
- Provide handout (available from *Thrombosis Canada*) describing new medication, atrial fibrillation, and follow-up; early renal function monitoring if new DOAC
- Do not initiate anti-arrhythmic agents like amiodarone or propafenone in the ED
- If sinus rhythm achieved, generally no need to initiate beta- or calcium channel-blockers

Box 1. Advisory committee members

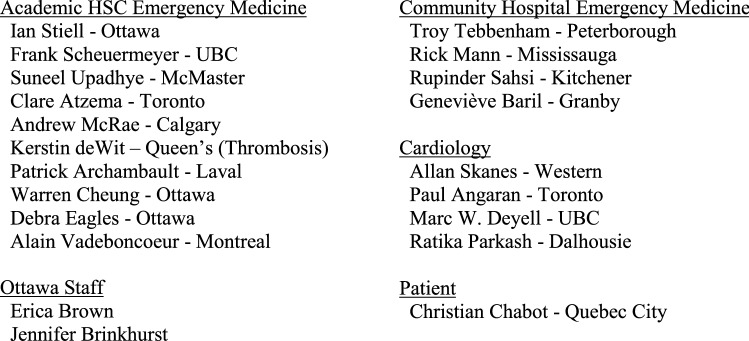

## Background and methods

The *2021 CAEP Acute Atrial Fibrillation/Flutter Best Practices Checklist* has been updated from the original version published in 2018 [1]. These checklists have been created to assist emergency physicians in Canada and elsewhere manage patients who present to the emergency department (ED) with acute/recent-onset atrial fibrillation (AF) or flutter (AFL). The checklist focuses on symptomatic patients with acute AF or AFL, i.e. those with recent-onset episodes (either first detected, recurrent paroxysmal or recurrent persistent episodes) where the onset is generally less than 48 h but may be as much as seven days. These are the most common acute arrhythmia cases requiring care in the ED. Canadian emergency physicians are known for publishing widely on this topic and for managing these patients quickly and efficiently in the ED [2, 3, 4].

**Fig. 1:**
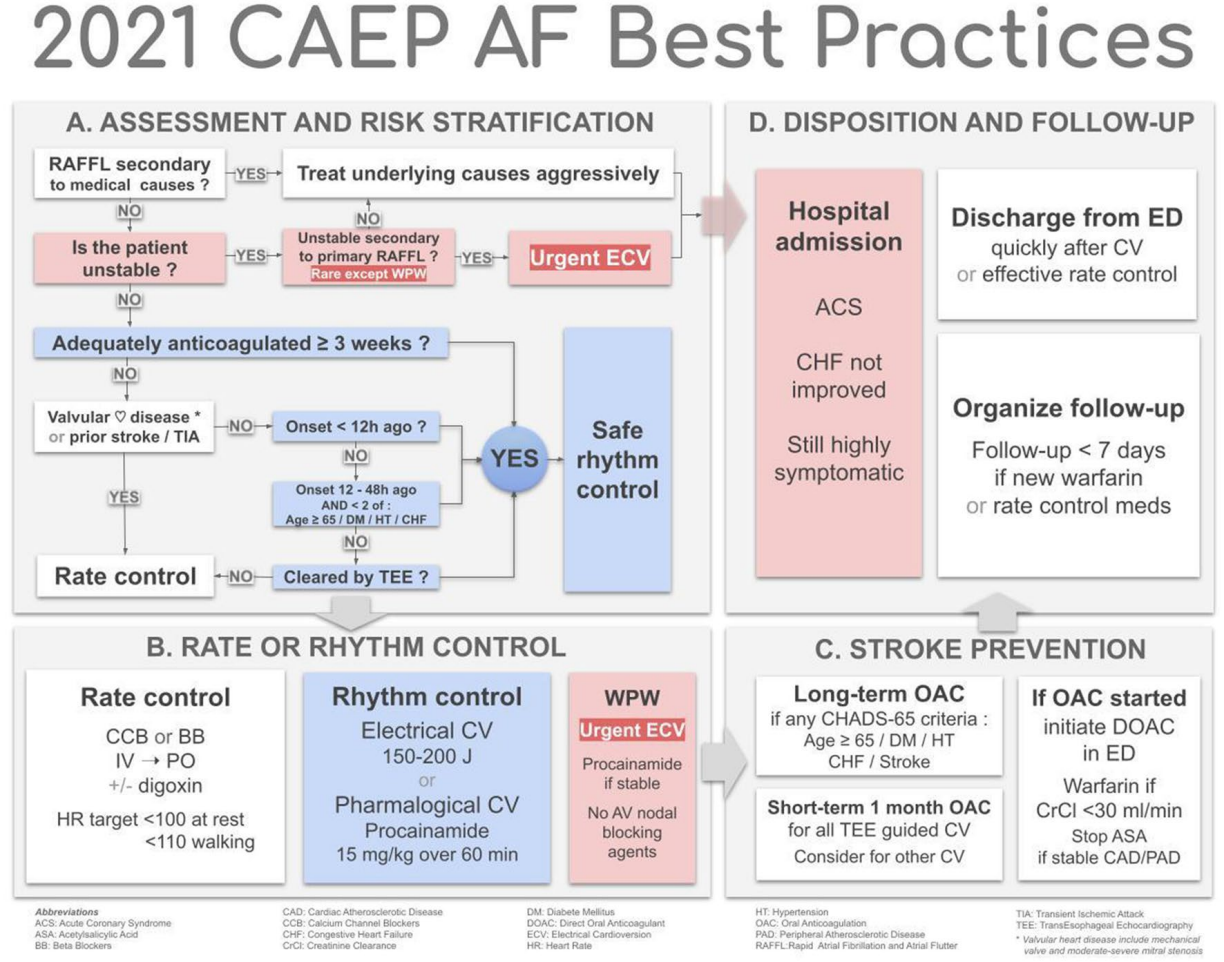
Overview of 2021 CAEP AF/AFL best practices checklist

The 2018 Checklist project was funded by a research grant from the Cardiac Arrhythmia Network and the resultant guidelines were formally endorsed by the Canadian Association of Emergency Physicians (CAEP). We chose to adapt, for use by emergency physicians, existing high-quality clinical practice guidelines (CPG) previously developed by the Canadian Cardiovascular Society (CCS) [5-7]. These CPGs were developed and revised using a rigorous process that is based on the GRADE (Grading of Recommendations Assessment, Development and Evaluation) system of evaluation [8]. With the assistance of our PhD methodologist (IG), we used the recently developed Canadian CAN-IMPLEMENT© process adapted from the ADAPTE Collaboration [9, 10]. We created an Advisory Committee consisting of ten academic emergency physicians (one also expert in thrombosis medicine), four community emergency physicians, three cardiologists, one PhD methodologist, and two patients. Our focus was four key elements of ED care: assessment and risk stratification, rhythm and rate control, short-term and long-term stroke prevention, and disposition and follow-up. The advisory committee communicated by face-to-face meetings, teleconferences, and email. The checklist was prepared and revised through a process of feedback and discussion on all issues by all panel members. These revisions went through ten iterations until consensus was achieved. We then circulated the draft checklist for comment to approximately 300 emergency medicine and cardiology colleagues. Finally, the CAEP Standards Committee posted the Checklist online for all CAEP members to provide feedback (Fig. 1).

Early in 2021 the same Checklist Advisory Committee reconvened (with one additional academic cardiologist) to discuss updates based upon new evidence [3, 4, 11], the 2018 and 2020 CCS guidelines [12, 13], and several commentaries that had expressed the concern of the Canadian ED community [14, 15]. The Advisory Committee met twice virtually and reached consensus on updates through repeated email exchanges. The panelists then sought further feedback from their own colleagues in emergency medicine and cardiology. Finally, the 2021 Checklist was posted by CAEP for further member feedback prior to final approval. The panel continues to believe that, overall, a strategy of ED cardioversion and discharge home from the ED is preferable from both the patient and the healthcare system perspective, for most patients. Many notable revisions were incorporated, including:

1. The safety of urgent cardioversion for acute AF/AFL depends upon anticoagulation status, prior stroke, valvular heart disease, time since onset, and CHADS criteria. Patients presenting between 12 and 48 h may only be cardioverted if they have 0 or 1 of the CHADS-65 criteria. We found that the CCS reference to CHADS_2_ Scale problematic as most ED physicians no longer use that scale.
2. Anticoagulation for CHADS-65 positive patients should be initiated in the ED unless there are contradictions as per the “McMaster Checklist” created by Dr. de Wit.
3. We disagree with the CCS suggestion of 4 weeks of anticoagulation for patients who are CHADS-65 negative as this was a weak recommendation per the GRADE system, based upon low quality evidence. We suggest that oral anticoagulation might be considered for a 4-week period after careful consideration of risks and benefits and a shared decision-making process with the patient.

Our hope is that the *2021 CAEP Acute Atrial Fibrillation/Flutter Best Practices Checklist* will standardize and improve care of AF and AFL in large and small EDs alike. We believe that these patients can be managed rapidly and safely, with early ED discharge and return to normal activities.
